# Unusual morphologies raise questions about the evolution of branching in kelps (Laminariales)

**DOI:** 10.1002/ece3.70109

**Published:** 2024-08-08

**Authors:** Samuel Starko

**Affiliations:** ^1^ UWA Oceans Institute and School of Biological Sciences Crawley Western Australia Australia

**Keywords:** adaptation, ecosystem engineers, evolution, foundation species, habitat, kelp, laminariales, morphology

## Abstract

Branching stipe morphologies have evolved multiple times across the kelp (Laminariales) lineage, creating morphological forms that drive the complexity of kelp forest habitats. Although branching is likely a complicated developmental process, it has evolved repeatedly through kelp evolution and the processes facilitating the emergence of branched forms from unbranched ancestors remain unclear. Here I report on abnormally branched individuals (*n* = 9) from five kelp species found in British Columbia, Canada that had atypical bifurcations in their stipes, creating a single dichotomous branch. One of these species generally lacks branching entirely (*Laminaria ephemera*) while the other four exhibit some branching but typically lack this stipe bifurcation (*Alaria marginata*, *Laminaria setchellii*, *Nereocystis luetkeana*, *Pterygophora californica*). These unusually branched individuals exhibited replicated morphological subunits distal to the stipe bifurcation, including more blades, pneumatocysts, and sporophylls than is typical. This suggests that unbranched species possess an inherent developmental capacity for modularity with autonomy in the development of individual modules that may have helped to facilitate the widespread emergence of branched morphologies. Given the role of kelp forests in coastal environments, branching may influence habitat characteristics, potentially influencing community dynamics, and is thus a trait of particular evolutionary interest. These findings highlight the need for experiments that manipulate kelp development to better characterise the ontogenetic processes of these globally important taxa.

## Graphical abstract

Branching stipe morphologies have evolved multiple times across the kelp lineage, influencing the complexity of kelp forest habitats. The author reports on anomalously branched individuals from five kelp species, exhibiting unusually replicated morphological subunits such as blades and floats. These anomalous morphologies may provide insights into how morphological innovations have evolved in kelps, and offer an important area for future research.

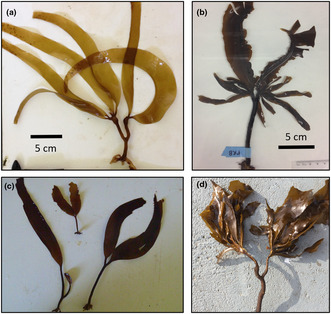

## 1 INTRODUCTION

The evolution and diversification of the kelps (Laminariales) reshaped coastal ecosystems as it unfolded over the past 30+ million years (Kiel et al., 2024; Starko et al., 2019; Vermeij et al., 2019). Kelps include some of the largest and most productive seaweed species found anywhere on earth, owed in part to specialised adaptations (e.g., internal vasculature) that facilitate rapid growth and sustain large sizes (Bringloe et al., 2020; Drobnitch et al., 2015; Graham et al., 2007; Jackson, 1987). Increased productivity in nearshore ecosystems has allowed other organisms to also grow larger and facilitated ecological feedbacks that selected for increased body size in a variety of coastal species (Vermeij, 2012; Vermeij et al., 2019). Like the invasion of embryophytes to land, the diversification and biogeographical spread of kelps across global oceans supplied nearshore ecosystems with large photosynthetic species that provide shelter and food energy for diverse assemblages of heterotrophic taxa (Duggins et al., 1989; Steneck et al., 2002; Teagle et al., 2017). These underwater forests are found across more than a third of the world's coastlines (Jayathilake & Costello, 2021; Starko et al., 2021) and hold tremendous ecological, economic, and cultural value (Eger et al., 2023). Despite their relatively recent appearance in nearshore ecosystems (Starko et al., 2019), kelps embody a fascinating diversity of morphologies that together create three‐dimensional forest communities (Steneck et al., 2002; Teagle et al., 2017) made more complex by this diversity of shapes and sizes.

**FIGURE 1:**
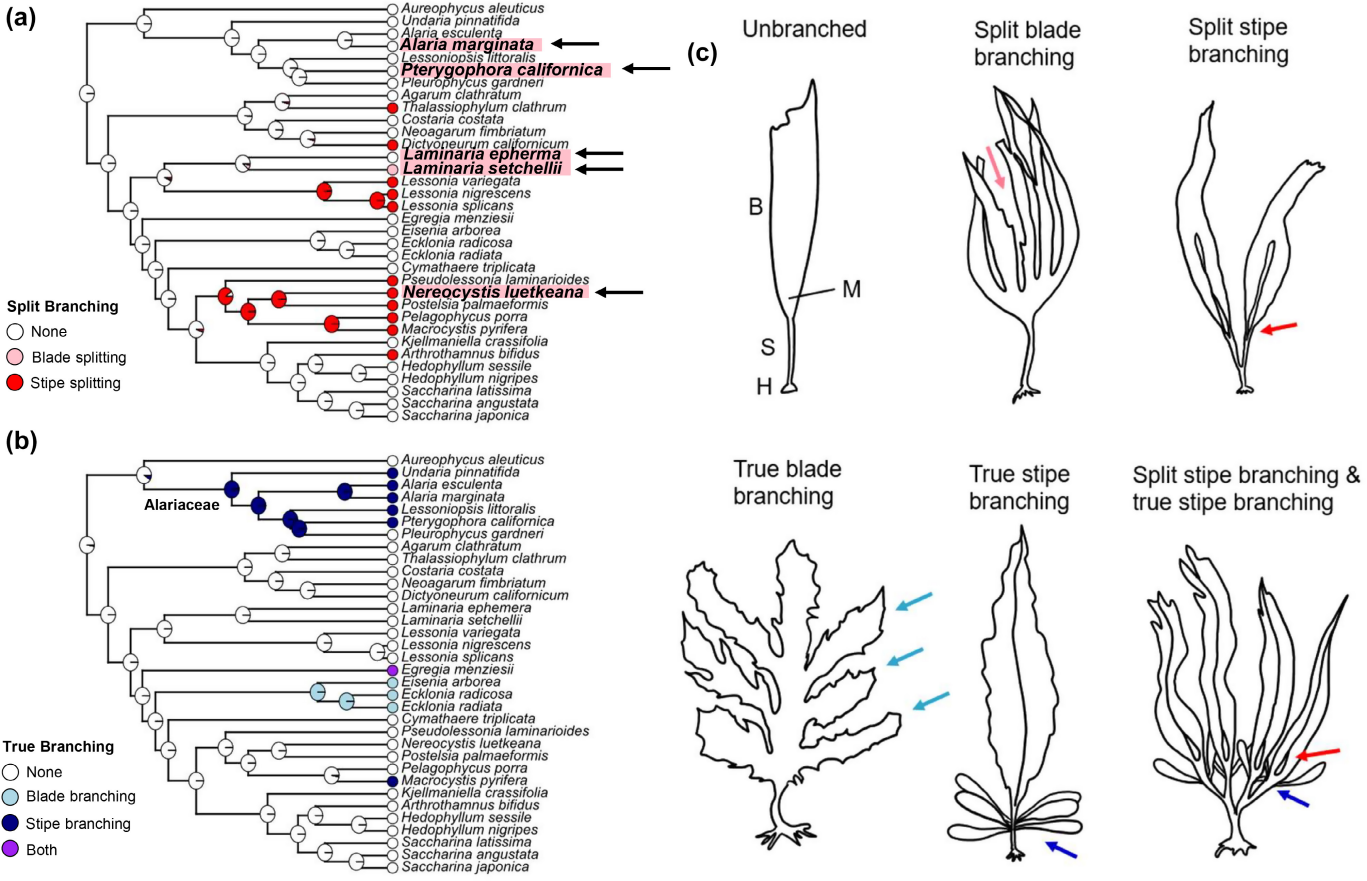
Distribution of branching across the kelp phylogeny with species discussed in this study. Ancestral state reconstruction of split branching (a) and true branching (b) across the kelp phylogeny (data and phylogenetic tree adapted from Starko et al., 2019). See Supplementary Information for methods. The common ancestor of the Alariaceae (which generally possess basal sporophylls) is specified in (b). Species from which unusually branched morphologies are reported in this study are highlighted in pink in (a) and indicated with a black arrow. (c) Diagrams of each feature shown in the phylogenetic reconstruction: An unbranched kelp (e.g., *Laminaria ephemera*), split blade (e.g., *Laminaria setchellii*) and stipe branching (*Lessonia variegata*), true blade (e.g., *Ecklonia radiata*) and stipe (e.g., *Alaria marginata*) branching, and a combination of both split and true branching (*Lessoniopsis littoralis*). The position of the holdfast (H), stipe (S), blade (B) and primary blade meristem (M) are indicated in the unbranched diagram of (c).

The kelp body plan is comprised of three key organs, the blade(s), stipe, and holdfast (Fritsch, 1935). While some growth occurs diffusely across the kelp thallus (e.g., Druehl, 1965; Kain et al, 1987; Kain, Norton et al., 1987), each organ generally has its own internal set of meristems where growth is maximal (Druehl, 1965; Kain et al, 1987; Wernberg et al., 2018). The primary, intercalary meristem is found at the base of the blade (i.e., closest to the stipe), while the stipe generally also has a meristem in its upper portion (i.e., closest to the blade) that facilitates stipe elongation (Druehl, 1965; Fritsch, 1935; Kain et al, 1987; Nicholson, 1970). Holdfasts generally possess multiple meristems at their haptera (Nicholson, 1970; Smith, 1939), analogous to root tips in plants, allowing the holdfast to grow and increase attachment area as the kelp grows larger (Nicholson, 1970; Starko & Martone, 2016). While some kelps possess other specialised morphological features (e.g., pneumatocysts), it is generally from these simple building blocks, that the diversity of forms observed across the kelp lineage have emerged. Branching is a conspicuous morphological trait that influences the complexity of kelp forest habitats (Wernberg et al., 2005) and is widespread across the kelps (Druehl et al., 1997; Starko et al., 2019) occurring in either the blade, the stipe or both. In general, branching in kelps can be conceptualised as one of two developmental processes (Figure 1): split branching (dissection of the primary meristem, usually via necrosis) and true branching (via additional lateral meristems). Split branching is the act of self‐dividing and is believed to involve programmed cell death or weakening of the tissues along the longitudinal axis of a kelp's blade, forming slits (MacMillan, 1899; Setchell, 1908; Setchell & Gardner, 1925; Wells, 1910). In most cases, these slits extend into the stipe, causing bifurcation and creating a branch. However, in some species, the slits only dissect the blade without extending into the stipe. It's important to note that passive, environmentally driven blade tearing or ripping (such as commonly observed in *Hedophyllum* spp.) is not considered split branching in this context. True branching, on the other hand, occurs in kelps that have secondary meristems (e.g., lateral meristems on the stipe). In the Alariaceae, this type of branching is associated with reproductive blades known as sporophylls that grow out from the stipe, proximal to the main blade (Setchell & Gardner, 1925). A small number of species (e.g., *Laminaria sinclarii*, *L. longipes*, *Macrocystis*) also undergo clonal growth through true branching along a rhizomatous holdfast (Fritsch, 1935; Murúa et al., 2017; Setchell & Gardner, 1925). These two branching mechanisms contribute to the diverse morphologies observed in kelp species, playing crucial roles in their growth patterns, reproductive strategies, and environmental adaptations.

Although all kelp species evolved from a simple, unbranched ancestor, both branched and unbranched morphologies are now found in every major kelp clade (Starko et al., 2019; Figure 1a,b). True branching and split branching have both evolved repeatedly with at least six separate origins of split branching and four origins of true branching (three stipe branching and one blade branching) inferred from ancestral state reconstruction (Figure 1a,b). Yet, with few exceptions (e.g., *Dictyoneurum* spp. may or may not branch via splitting) the presence or absence of branching is generally fixed at the level of species (or genus) and it remains unclear what genetic or developmental processes are required to evolve branching from an unbranched ancestor. Here, I report on several unusually branched individuals from five kelp species. While these are unlikely to be genetic mutants, their existence in nature poses questions requiring future study and may shed light on developmental processes involved in branching. Specifically, these forms suggest a level of inherent modularity (sensu Harper, 1977, 1981) in all kelps (including unbranched taxa) which may have facilitated the parallel emergence of complex, i morphologies. Future research is needed to better understand the cause of these unusual morphologies and their significance in kelp evolution.

## 2 RESULTS & DISCUSSION

**FIGURE 2:**
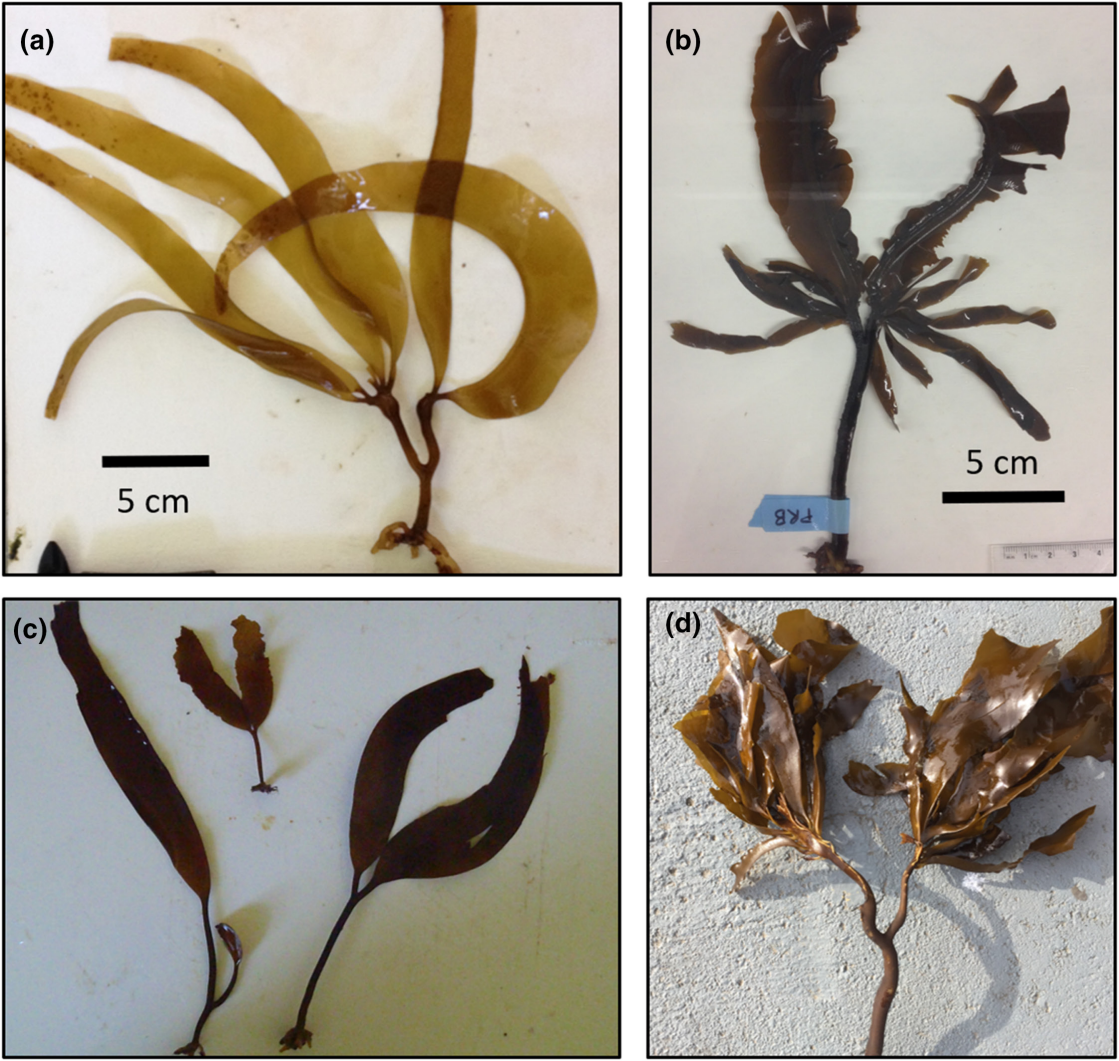
Examples of unusually branched kelp specimens. (a) *Nereocystis luetkeana*, (b) *Alaria marginata*, (c) *Laminaria setchellii*, and (d) *Pterygophora californica* collected from around Vancouver Island between 2014 and 2020.

Over the past decade, I have found several individuals of five species (Figure 2, Data S1; Table 1) in British Columbia, Canada that had obvious branching in their stipes atypical of their species. I recorded individuals of *Laminaria ephemera* (*n* = 1; Figure S1), *Laminaria setchellii* (*n* = 4; three from the same site; Figure 1c; Figure S2), *Alaria marginata* (*n* = 1; Figure 1b), *Pterygophora californica* (*n* = 2; Figure 1d) and *Nereocystis luetkeana* (*n* = 1; Figure 1a) that each had single unusual dichotomies in their stipes. Both *L. ephemera* and *L. setchellii* generally lack stipe branching with blade splitting present in *L. setchellii* but no branching at all is known from *L. ephemera* (Figure 1). In contrast, *N*. *luetkeana*, *A*. *marginata* (Figure 2b), and *P*. *californica* (Figure 1d), are all multibladed, despite typically lacking this dichotomy. *A. marginata* and *P. californica* have sporophylls that grow as true branches from both sides of their stipe, while *N. luetkeana* has multiple blades arising from a terminal pneumatocyst (gas‐filled floats) at the end of its stipe. While *N. luetkeana* does have split branching into the stipe (Figures 1 and 2), this typically only bifurcates small portions of the stipe above the pneumatocyst and therefore does not cause branching in the main stipe axis (below the pneumatocyst). In all unusual specimens (*n* = 9), the dichotomous branch occurred well below the main blade and the entire structure distal to the branch was replicated as a single unit. Distal to the branch in *N. luetkeana* were two pneumatocysts with a set of blades arising from each (Figure 2a). For *A. marginata* and *P. californica*, each branch bore sporophylls on both sides of the stipe and terminated with a blade and midrib, as is characteristic of the Alariaceae. Except for one *P. californica* individual which was more than a metre long and the *L. ephemera* individual which had soral patches, these unusual individuals were generally in the juvenile stage. Unusually branched morphologies have been observed by others (e.g., Calloway et al., 2020, L. Druehl, personal communication, T. Mumford, personal communication) and are even featured in First Nations mythology (Calloway et al., 2020). However, the insights that these morphologies potentially shed on morphogenesis in kelp has not been discussed in the literature.

**TABLE 1:** Information on the observations of unusual kelp morphologies.

Species	Family	Site	Coordinates	Date	Relevant figure
*Alaria marginata* (*n* = 1)	Alariaceae	Prasiola Point, Barkley Sound	48.8176 N, −125.1686 W	October, 2014	Figure 1b
*Pterygophora californica* (*n* = 1)	Alariaceae	Ogden Point, Victoria	48.4130 N, −123.3879 W	May 2017	Figure 1d
*Pterygophora californica* (*n* = 1)	Alariaceae	North Beach, Calvert Island	51.6657 N, −128.1353 W	June 2018	Figure S3
*Laminaria ephemera* (*n* = 1)	Laminariaceae	Edward King Island, Barkley Sound	48.8256 N, −125.2120 W	June, 2015	Figure S1
*Laminaria setchellii* (*n* = 3)	Laminariaceae	Scott's Bay, Barkley Sound	48.8344 N, −125.1472 W	July, 2015	Figure 1c
*Laminaria setchellii* (*n* = 1)	Laminariaceae	Edward King Island, Barkley Sound	48.8366 W, −125.2136 N	July 2020	Figure S2
*Nereocystis luetkeana* (*n* = 1)	Arthrothamnaceae	Scott's Bay, Barkley Sound	48.8344 N, −125.1472 W	June, 2015	Figure 1a

While the developmental processes that produced these unusual morphologies are not entirely clear, they most likely arose through a process analogous to split branching. Split branching in kelps occurs by means of developmental splitting of the meristematic regions of the blade and stipe (Druehl et al., 1997; MacMillan, 1899; Setchell & Gardner, 1925) leading to replication of part of the kelp (Figure 1). Generally, select cells in the photosynthetic surface layer (meristoderm) will undergo programmed cell death, followed by growing in of the edges to form a branch (Druehl et al., 1997, Druehl pers comm). I hypothesise that the unusual specimens I describe here arose through physical damage to the primary blade meristem (and into the stipe proximal to it) that artificially mimicked the process of developmental splitting. Indeed, some deformation in the stipe (small bumps) were obvious in a few samples (*A*. *marginata*, both *P*. *californica*), possibly indicating damage. Moreover, in a few of the specimens (*A*. *marginata*, *N*. *luetkeana*, and two *L*. *setchellii*), the subunits on either side of the bifurcation (e.g., blades) were asymmetrical in shape and size with the side of each blade closest to the branch appearing to have delayed development which might reflect damage (i.e., tearing; Figure 2a–c; Figure S2). For *L. setchellii* and *N. luetkeana*, splitting already occurs naturally and a tear of one of these slits deeper into the meristem and stipe than is normal could explain these unusual morphologies. Some specimens had smooth stipes throughout, however, including at the dichotomy, and were from species that exhibit no natural blade splitting. Thus, genetic mechanisms for some of these specimens cannot be completely ruled out. However, all unusual specimens were found in wave exposed environments, potentially making them prone to damage during early development. Future experimentation could be conducted to simulate meristematic damage (e.g., cutting a slit in the meristem to replicate tissue splitting) and determine whether branching can be induced through exogenous factors. Another possible cause of these morphologies is that these each reflect two chimeric individuals, such as those found in *Lessonia* spp. (e.g., González & Santelices, 2017) or from *Macrocystis* gametophytes with multiple oogonia (Buschmann et al., 2020). This is unlikely however, since chimeric kelps are known to share a fused holdfast but not large portions of stipe as would be necessary to produce these specimens.

Split branching that extends into the stipe generally leads to the replication of morphological subunits distal to the split, and this is one of the key mechanisms producing highly branched kelp morphologies (Druehl et al., 1997; Starko et al., 2019). For example, *Lessoniopsis littoralis* is the only member of the Alariaceae that exhibits split branching and, like *A*. *marginata* and *P*. *californica*, has a central blade with adjacent sporophylls (produced as true branches via lateral meristems on the stipe). However, this entire set of blades is replicated many times over through repeated splitting of the central blade into upper portions of the stipe, leading to a highly branched and arborescent morphology (Figure 1h depicts this species). The observation of branched *A. marginata* and *P. californica* (also from Alariaceae) specimens with replicated sporophyll‐blade subunits reminiscent of *Lessoniopsis littoralis*, suggests that even unbranched kelps possess the inherent capacity to replicate subunits of their body‐plans if upper portions of the stipe split or are torn. This capacity for meristematic tissue to produce structures distal of it as a single module, potentially makes the evolution of split branching somewhat straightforward. For example, the evolution of successive splitting in the meristematic region (e.g., through a novel mutation) would produce highly branched, indeterminantly growing individuals with seemingly complex morphologies without requiring additional evolutionary steps. Branch patterns and timings could then be reshaped through natural selection.

Splitting of tissues by means of programmed cell death is common characteristic, even in some unbranched kelps. For example, *L*. *setchellii*, one of the species found to contain anomalously branched individuals normally produces a dissected blade with splitting occurring part way down the blade. Similarly, *N. luetkeana* exhibits ontogenetic blade splitting that produces many blades with distinct meristems (Nicholson, 1970), and uses programmed cell death to release its deciduous soral patches (Walker, 1980). Some species from the Agaraceae also use programmed cell death to produce blade fenestrations (Humphrey, 1886), despite an overall lack of split branching. Given that kelps possess an inherent capacity for modularity and the physiological machinery to undergo splitting (e.g., cell necrosis) is widespread and likely evolutionarily labile across the kelps, it is perhaps not surprising that branching has been lost and gained so frequently through the diversification of kelps (Figure 1a,b). The signalling mechanisms involved in natural tissue splitting and the underlying genetic drivers remain unclear but growing evidence suggests that hormones play a key role in morphogenesis of kelps in general (e.g., Boscq et al., 2024).

Growth by indeterminant replication of subunits is common in plants and colonial animals and it is believed that the dynamics that govern the ecology and evolution of these organisms may be fundamentally different from those that influence unitary, determinant lifeforms, such as vertebrates (Harper, 1977, 1981; Vuorisalo & Mutikainen, 2001). Interestingly, unbranched kelp species provide an intermediate form between these two extremes. Although single‐bladed kelps grow indeterminately by means of an intercalary meristem (Fritsch, 1935), the number of subunits that make up their body‐plan is generally fixed to only one of each organ (blade, stipe, and holdfast). The presence of these branched individuals among populations with fixed morphologies suggests that the machinery required to grow by replication of subunits (i.e., to achieve modularity) is widespread. Indeed, recent evidence that frond growth in the highly branched *Macrocystis* may be determinate to some extent (Stephens, 2017) supports the notion that some taxa, like kelps, lie in grey area between unitary and truly modular organisms.

The morphology of habitat‐forming species can have fundamental bottom‐up effects on the communities that they help shape and establish (Jones et al., 1997; Lawton & Jones, 1995; Teagle et al., 2017); kelp forests exemplify this phenomenon (Steneck et al., 2002; Teagle et al., 2017). Kelp communities can contain multiple layers of canopy and understory, creating intricate habitat with many levels for use by other organisms. Branching may be of particular importance, since it can influence both the way that water moves through a habitat (Hurd, 2000; Wernberg et al., 2005), and the complexity of habitats for associated species (Teagle et al., 2017; Wernberg et al., 2005). The existence of these anomalous morphologies may offer some insights into the processes by which branching occurs during development and highlights an important area for future research.

## AUTHOR CONTRIBUTIONS

**Samuel Starko:** Conceptualization (lead); investigation (lead); writing – original draft (lead); writing – review and editing (lead).

## Supporting information


Data S1.

## Data Availability

All data are made available in the supplementary information or main text of this article.
