# Supplementary material for: Unusual morphologies raise questions about the evolution of branching in kelps (Laminariales)
Source: Ecol Evol. 2024 Aug 8;14(8):e70109. doi: 10.1002/ece3.70109 (PMC11310088; doi:10.1002/ece3.70109)
Supplement: Supplementary file 1 — Data S1. [file ECE3-14-e70109-s001.docx]

**Supplementary Information**

**Supplementary Methods**

To reconstruct the evolution of branching across the kelp phylogeny, I used a likelihood based ancestral state reconstruction analysis. I used the time-calibrated phylogeny from Starko et al. (2019) but replaced the *Laminaria hyperborea/L. digitata* clade with *L. setchellii* which is appropriate based on genus-level phylogenies (Rothman et al., 2017). I used trait values from Starko et al. (2019) that are mostly based on Setchell & Gardner (1925). Pseudo-branching of the stipe, such as the forked stipe of *Eisenia arborea* was considered special cases of blade branching since the branch forms when the tissue is a blade and then it becomes part of the stipe as the kelp grows and develops. Ancestral state reconstruction was conducted in R, using the packages “ape” and “phytools”.

**Supplementary References**

Rothman, M. D., Lydiane, M., Anderson, R. J., Bolton, J. J., & Verbruggen, H. (2017). A phylogeographic investigation of the kelp genus *Laminaria* (Laminariales, Phaeophyceae), with emphasis on the South Atlantic Ocean. *Journal of Phycology*, *53*(4), 4. https://doi.org/10.1111/jpy.12544

Setchell, W. A., & Gardner, N. L. (1925). *The Marine Algae of the Pacific Coast of North America: Malanophyceae*. University of California Press.

Starko, S., Soto Gomez, M., Darby, H., Demes, K. W., Kawai, H., Yotsukura, N., Lindstrom, S. C., Keeling, P. J., Graham, S. W., & Martone, P. T. (2019). A comprehensive kelp phylogeny sheds light on the evolution of an ecosystem. *Molecular Phylogenetics and Evolution*, *136*, 138–150. https://doi.org/10.1016/j.ympev.2019.04.012


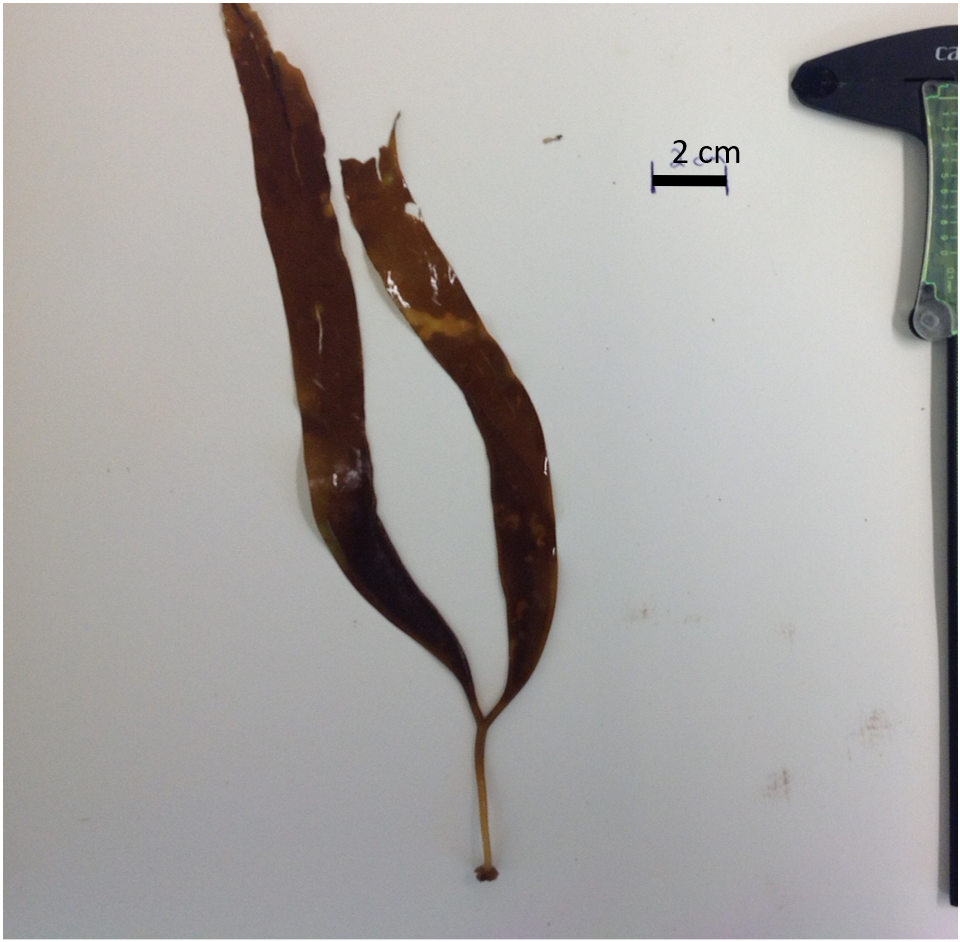


**Fig S1. A branched individual of *Laminaria ephemera* collected on Edward King Island, Barkley Sound.**


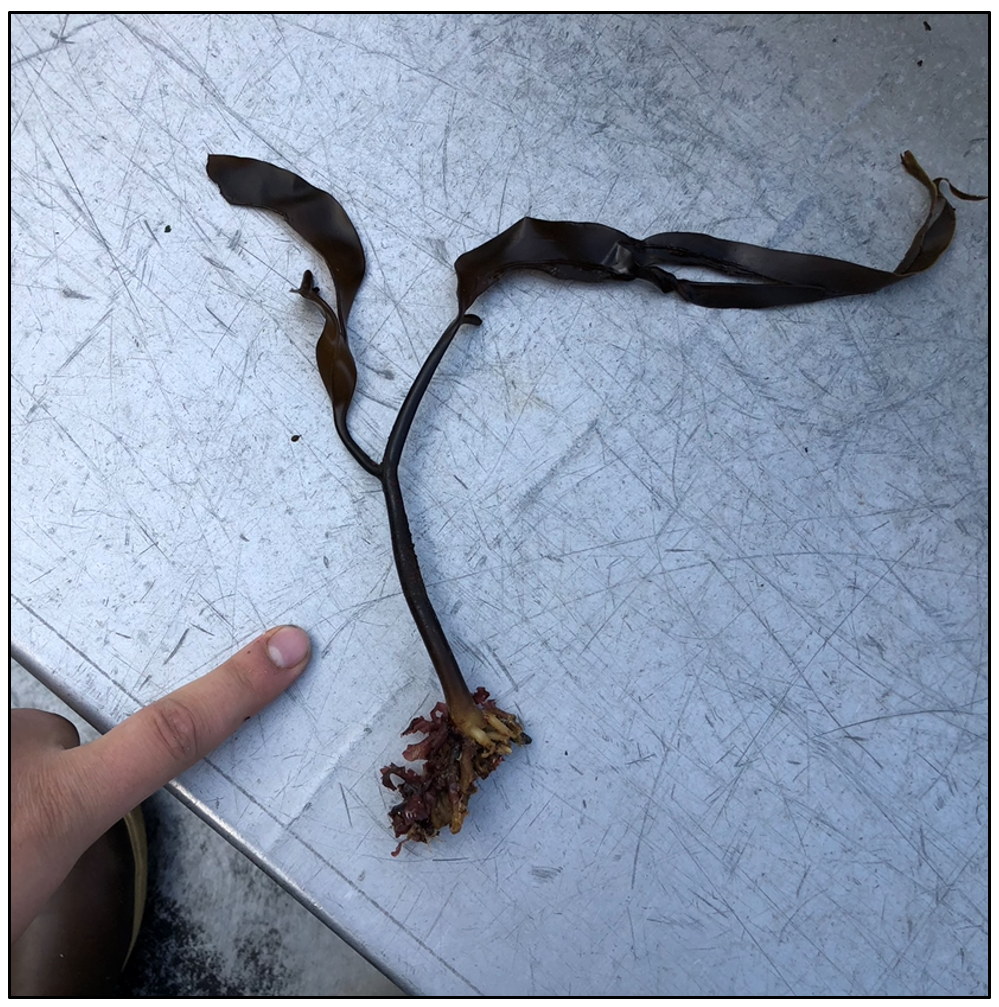


**Fig S2. A branched individual of *Laminaria setchellii* collected on Edward King Island, Barkley Sound.**


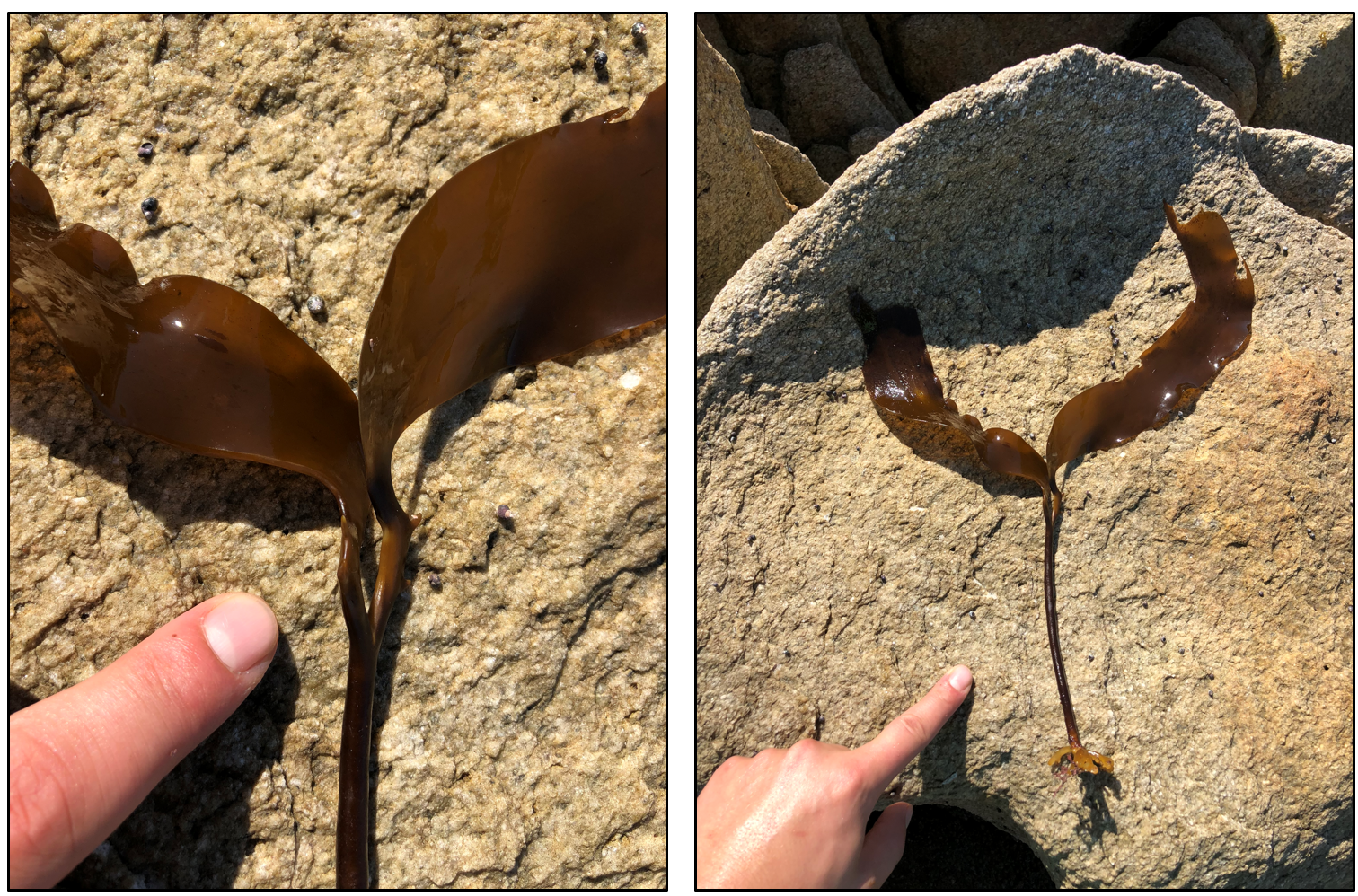


**Fig S3. A branched individual of *Pterygophora californica* collected at North Beach, Calvert Island.**
